# Macrophages and cancer stem cells: a malevolent alliance

**DOI:** 10.1186/s10020-021-00383-3

**Published:** 2021-09-28

**Authors:** Paola Allavena, Elisabeth Digifico, Cristina Belgiovine

**Affiliations:** Present Address: Humanitas Clinical and Research Center -IRCCS, via Manzoni 56, 20089 Rozzano, MI Italy

**Keywords:** Cancer stem cells, Tumor associated macrophages, Stemness, GPNMB

## Abstract

Myeloid cells infiltrating tumors are gaining ever growing attention in the last years because their pro-tumor and immunosuppressive functions are relevant for disease progression and therapeutic responses. The functional ambiguity of tumor-associated macrophages (TAMs), mostly promoting tumor evolution, is a challenging hurdle. This is even more evident in the case of cancer stem cells (CSCs); as active participants in the specialized environment of the cancer stem cell niche, TAMs initiate a reciprocal conversation with CSCs. TAMs contribute to protect CSCs from the hostile environment (exogenous insults, toxic compounds, attacks from the immune cells), and produce several biologically active mediators that modulate crucial developmental pathways that sustain cancer cell stemness. In this review, we have focused our attention on the interaction between TAMs and CSCs; we describe how TAMs impact on CSC biology and, in turn, how CSCs exploit the tissue trophic activity of macrophages to survive and progress. Since CSCs are responsible for therapy resistance and tumor recurrence, they are important therapeutic targets. In view of the recent success in oncology obtained by stimulating the immune system, we discuss some macrophage-targeted therapeutic strategies that may also affect the CSCs and interrupt their malevolent alliance.

## Background

Macrophages are cells of the innate immunity belonging to the mononuclear phagocyte system. Tissue-resident macrophages originate from embryonic progenitors in the yolk sac and fetal liver, and seed peripheral organs to become specialized macrophages, such as liver Kuppfer cells, brain microglia, lung alveolar macrophages and bone osteoclasts. They function to maintain homeostasis and to limit the entrance of pathogens (Gordon and Pluddemann 2019; Wynn et al. 2013; Yona et al. 2013). Blood circulating monocytes, originating from hematopoietic bone marrow precursors, can be recruited at peripheral tissues upon inflammatory conditions or tissue damage, and differentiate into mature macrophages (Gordon and Pluddemann 2019; Wynn et al. 2013; Yona et al. 2013). Both resident and recruited macrophages are characterized by a high grade of phenotypic and functional plasticity that is dictated by distinct genetic programs, triggered by specific local stimuli, such as the granulocyte–macrophage growth factor (GM-CSF), macrophage growth factor (M-CSF), Th1 and Th2 cytokines (Gordon and Pluddemann 2019; Wynn et al. 2013; Yona et al. 2013; Mantovani et al. 2017; Biswas 2015). Their broad spectrum of activation states can be simplified by defining the two extreme functional phenotypes, popularly named M1 and M2 macrophages (Mills et al. 2000; Mantovani et al. 2002; Murray et al. 2014). M1 or classically activated macrophages are typically stimulated by IFN γ, or by the engagement with bacterial components (e.g., lipopolysaccharides, LPS); they produce pro-inflammatory cytokines, such as IL-1β, TNFα, IL-12. M1 macrophages actively counteract bacterial infections and stimulate the activation of adaptive immune cells. On the other extreme, M2 or alternatively activated macrophages have distinct and sometimes opposite functions, being responsible for the suppression of Th1 immune responses, the promotion of tissue healing and the resolution of inflammation. M2 macrophages are activated by anti-inflammatory cytokines, such as IL-4 and IL-13, and are also affected by the immunosuppressive mediators IL-10 and Transforming Growth Factor β (TGFβ). In physiological conditions, M1 and M2 macrophages are two essential players that regulate the balance between active immune responses and homeostasis (Gordon and Pluddemann 2019; Murray et al. 2014; Pollard 2009; Mantovani et al. 2013).

In the tumor context they also differ, as M1 macrophages inhibit tumor progression by directly killing cancer cells and promoting anti-tumor immune responses, while M2 macrophages stimulate angiogenesis and tumor growth (Mantovani et al. 2017; Allavena et al. 2021; Belgiovine et al. 2020).

Tumor-associated macrophages (TAMs) are key players in the tumor microenvironment and frequently represent the most abundant immune population (Mantovani et al. 2017). They are profoundly conditioned by the presence of tumor cells and acquire, most commonly, an M2-like phenotype. It is now overall accepted that TAMs promote tumor progression, as they actively enhance cancer cell proliferation and strongly suppress anti-tumor immune responses (Mantovani et al. 2017; Allavena and Mantovani 2012; Palma and Lewis 2013; Belgiovine et al. 2016; Quaranta and Schmid 2019). In particular, TAMs produce a large array of soluble mediators to support tumor cell proliferation, such as Epidermal Growth Factor (EGF), Platelet-derived Growth Factors (PDGF) and Vascular Endothelial Growth Factor (VEGF). TAMs also produce several cytokines that have immunosuppressive activity on other immune cells: IL-10 and TGFβ (Mantovani et al. 2013, 2017; Akiko Kogure and Ochiya 2019; Biswas et al. 2013). Furthermore, TAMs actively produce several proteolytic enzymes, such as matrix metalloproteases, serine proteases and cathepsins, thereby enhancing the remodeling of the tumor stroma and favoring the metastatic process of cancer cells (Liguori et al. 2011; Zhang et al. 2017; Sangaletti et al. 2014; Chen et al. 2011; Steenbrugge et al. 2018; Wang et al. 2011,2018; Aras and Zaidi 2017). While it is well established that TAMs display these supporting functions on the proliferating cancer cells, relatively few studies have addressed the impact of TAMs on the specific population of tumor-initiating cells or Cancer Stem Cells (CSCs) (Chen et al. 2021; Raggi et al. 2016; Aramini et al. 2021; Fan et al. 2014; Jinushi et al. 2011; Osman et al. 2020).

CSCs cells are cellular elements in the tumor tissue with stem-like properties which have been demonstrated to play a key role in disease progression and tumor recurrence. They represent a distinctive cell subset within the tumoral mass and are characterized by unlimited self-renewal properties, tumor initiation ability and chemo-resistance (Kreso and Dick 2014; Nguyen et al. 2012). The existence of CSCs has been reported in several tumor types, including breast cancer (Al-Hajj et al. 2003), lung cancer (Eramo et al. 2006), acute myeloid leukemia (Lapidot et al. 1994), pancreatic cancer (Li et al. 2007; Hermann et al. 2007), hepatocellular carcinoma (Miranda-Lorenzo et al. 2014), head and neck cancer (Prince et al. 2007), colon cancer (O'Brien et al. 2007; Ricci-Vitiani et al. 2007), melanoma (Schatton et al. 2008; Quintana et al. 2008), prostate cancer (Patrawala et al. 2006), and glioblastoma (Singh et al. 2004). CSCs can be defined in vitro in functional assays (i.e., tumor-sphere assay), as cells with intrinsic drug resistance and self-renewal potential but are most commonly defined by the expression of stemness-related markers. CSCs-associated markers include: aldehyde dehydrogenase 1 (ALDH1), involved in intracellular retinoic acid production; ATP-binding cassette sub-family G member 2 (ABCG2); other surface markers such as CD133, CD44, CD24, CD34, CD90, CD117 and CD166 (Fan et al. 2014; Jinushi et al. 2011; Kreso and Dick 2014; Hermann et al. 2007; Ginestier et al. 2007; Medema 2013; Hanahan and Coussens 2012; Hsu and Fuchs 2012; Korkaya et al. 2011; Liguori et al. 2021; Lu et al. 2014; Plaks et al. 2015; Zhou et al. 2015).

Here we review the available scientific literature about the interaction of TAMs with CSCs; we present how TAMs support cancer cell stemness and, in turn, how CSCs exploit the presence and pro-tumor functions of macrophages to survive and progress; finally we discuss how recent therapeutic approaches directed to macrophage may impact on CSCs and interrupt their deleterious dialogue.

## Main text

### The specialized environment of the stem cell niche

In normal tissues stem cells reside in a specialized environment, the stem cell niche, where they are protected from exogenous insults and receive from nearby cells the necessary factors for their survival and maintenance of their stemness status (Lu et al. 2014; Plaks et al. 2015).

The stem cell niche is populated by different cell types, such as stromal mesenchymal cells (fibroblasts, activated myofibroblasts), immune cells (especially macrophages) blood and lymphatic vessels; the niche is embedded in a scaffold of extracellular matrix (ECM) molecules composed by collagenous fibers, proteoglycans and several glycoproteins, e.g. laminin, fibronectin, tenascin-C, Secreted Protein Acidic and Rich in Cysteine (SPARC), periostin (POSTN) and other (Nallanthighal et al. 2019).

This specialized tissue structure constitutes the ideal environment where stem cells can survive and remain quiescent; the niche, however, may also provide cues for stem cell proliferation, differentiation and migration. These processes are initiated and regulated by several molecular pathways, including cytokines and growth factors (e.g. IL-6, TGFβ), signaling receptors (CD44, Notch family receptors and their cognate ligands), and specific transcription factors (Sonic Hedgehog—SHH, SOX2, OCT3/4 and NANOG (Plaks et al. 2015; Pickup et al. 2014; Clara et al. 2020).

In tumors, also the CSCs reside in a cancer niche that defends them from stress signals, such as apoptosis-inducing chemotherapeutic agents and from attacks by the immune system (Hanahan and Coussens 2012; Hsu and Fuchs 2012; Korkaya et al. 2011; Plaks et al. 2015). As detailed below, key players in the cancer niche are TAMs, which indeed secrete a variety of soluble factors and physically interact with CSCs to protect them from environmental damage (Fan et al. 2014; Jinushi et al. 2011; Liguori et al. 2021; Lu et al. 2014; Zhou et al. 2015; Oshimori 2020; Raghavan et al. 2019).

### The macrophage-CSC crosstalk

It is well established that macrophages in peripheral tissues have trophic activity for the nearby cells and contribute to preserve the physiologic homeostasis (Gordon and Pluddemann 2019; Wynn et al. 2013). Macrophages play a crucial role also in the development and morphogenesis of different organs during the embryonic life (Gordon and Pluddemann 2019; Wynn et al. 2013; Mantovani et al. 2013). This activity is mainly reflected in their ability to protect and support organ progenitor stem cells, for instance during the development of the ductal epithelial tree of the mammary gland (Gyorki et al. 2009; O'Brien et al. 2010). This tissue trophic ability occurs also in the tumor tissue where infiltrated TAMs initiate a reciprocal crosstalk with cancer cells that eventually results in enhanced tumor cell survival and disease progression. A well-known example is the paracrine loop occurring between breast cancer cells, producing the myeloid differentiation factor M-CSF, and macrophages which, in turn, release EGF to sustain cancer cell proliferation (Wyckoff et al. 2004).

#### From the cancer stem cell side

**Fig. 1:**
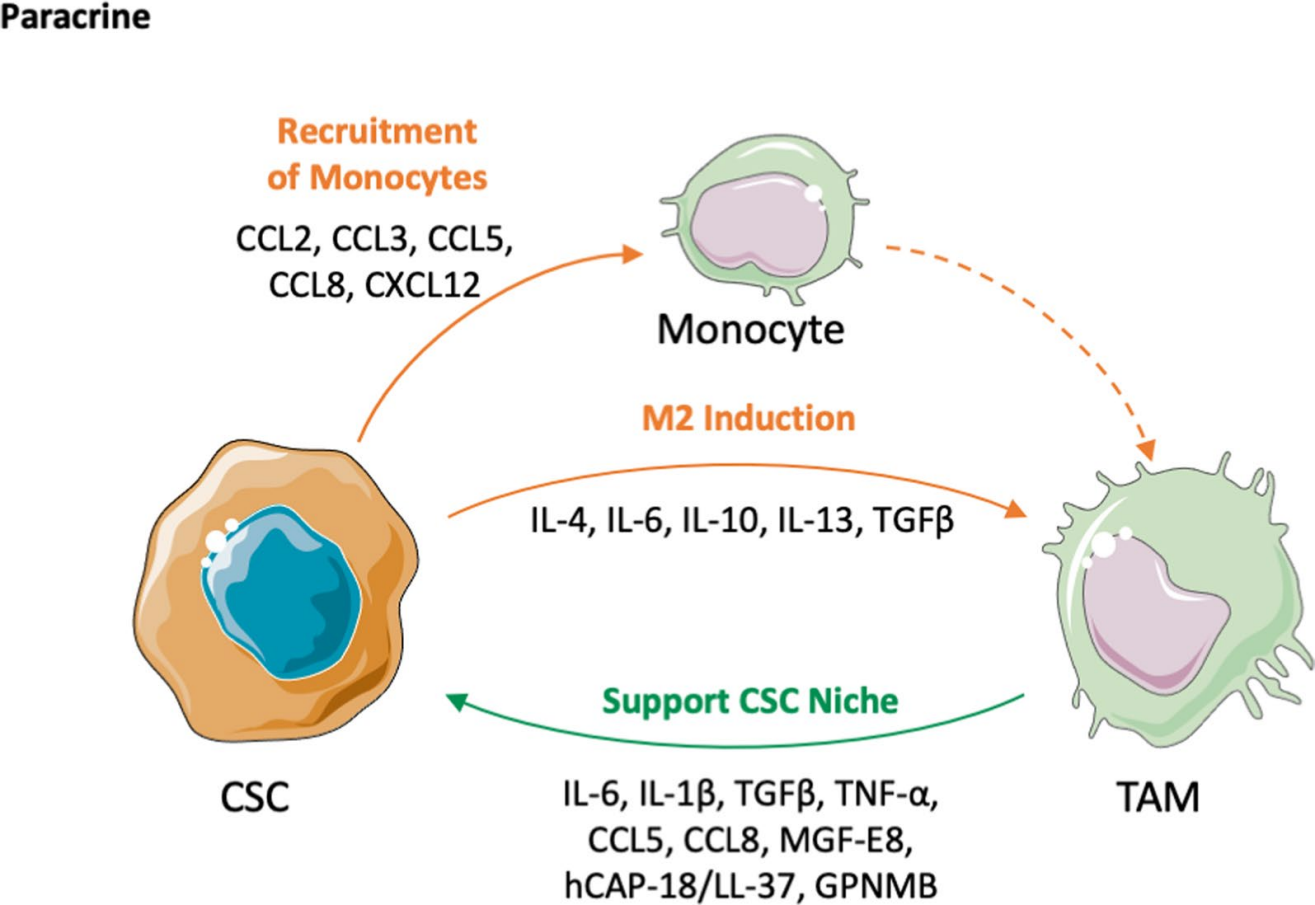
Paracrine mechanisms of interaction between cancer stem cells (CSCs) and tumor-associated macrophages (TAM). CSCs produce chemotactic factors (CCL2, CCL3, CCL5, CCL8, CXCL12) to recruit circulating monocytes in the tumor; moreover, they shape macrophage polarization towards an M2-like, pro-tumoral, phenotype by secreting IL-4, IL-6, IL-10, IL-13, TGFβ. On the macrophage side, TAMs support CSCs and their niche by secreting IL-6, IL-1β, TGFβ, TNF-α, CCL5, CCL8, MGF-E8, hCAP-18/LL-37, GPNMB. This figure was made with Servier Medical Art templates, which are licensed under a Creative Commons Attribution 3.0. Unported License (https://smart.servier.com)

**Fig. 2:**
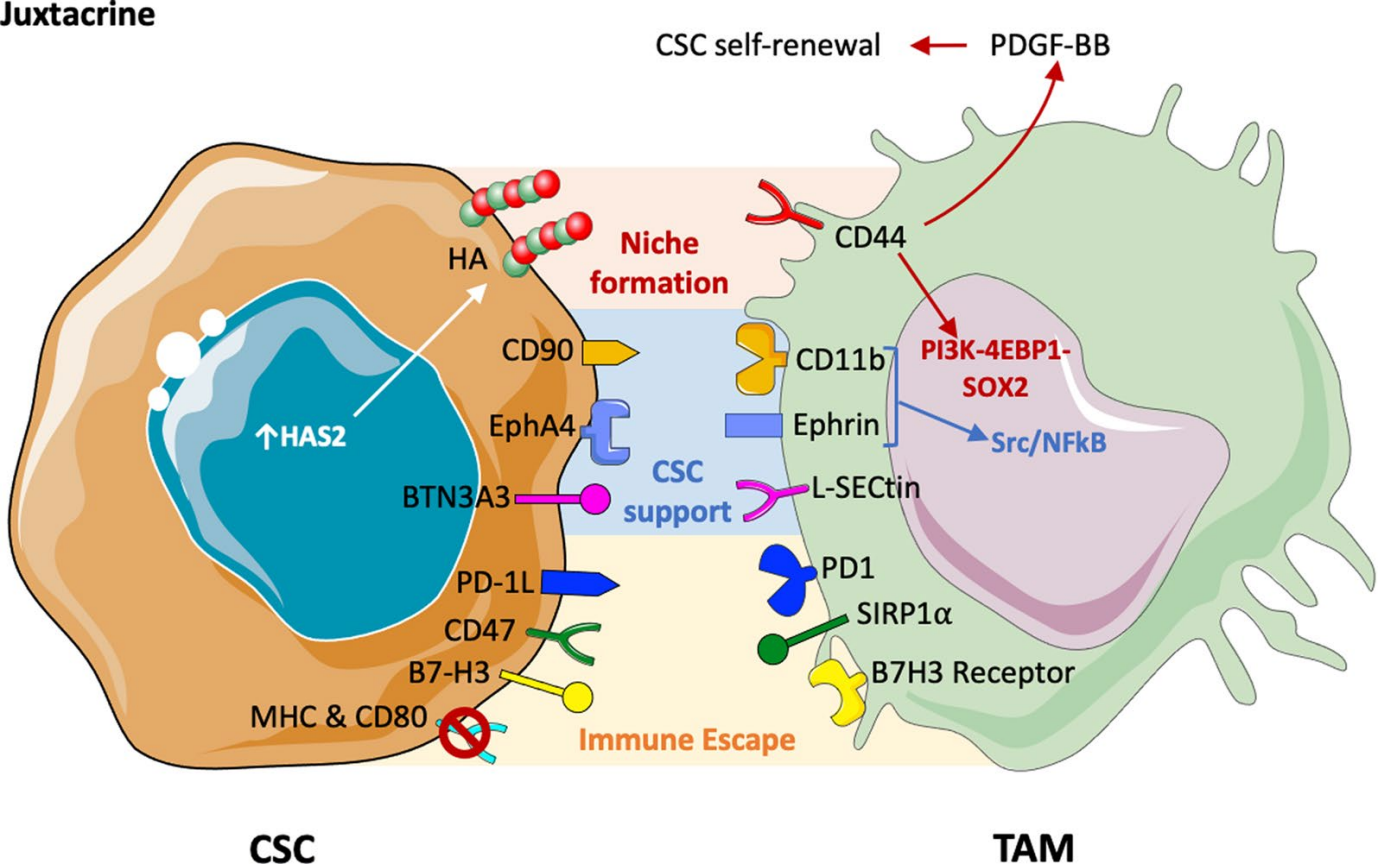
Juxtacrine mechanisms of interaction between cancer stem cells (CSCs) and tumor-associated macrophages (TAM). CSCs upregulate the enzyme hyaluronan synthase 2 (HAS2), which induces the formation of a layer of pericellular hyaluronan (HA) that facilitates their attachment to TAMs, via the CD44 receptor. Upon this interaction, TAMs produce the growth factor PDGF-BB, which enhances CSC self-renewal; moreover, it activates signaling pathways important for CSC maintenance (e.g.PI3K–4EBP1–SOX2). CSCs express CD90 and the receptor EphA4, which bind to CD11b and Ephrin, respectively. These interactions activate the Src/NFkB pathway, and together with the LSECtin and BTN3A3 they support and drive cancer stemness. Finally, CSCs downregulate MHC molecules and CD80, while upregulating PD-1L, CD47, B7-H3 to escape from the immune system recognition. This figure was made with Servier Medical Art templates, which are licensed under a Creative Commons Attribution 3.0. Unported License (https://smart.servier.com)

To take advantage of the trophic activity of macrophages, CSCs—like most cells constituting a tumor—produce chemotactic factors that recruit macrophage precursors (circulating monocytes), as well as tissue macrophages resident in the nearby area. Among the various chemokines, evidence has been provided that chemokine CCL2, CCL3, CCL5, CCL8 and CXCL12 actively participate in this process (Chen et al. 2019a, 2021; Zhou et al. 2015; Zeng et al. 2018; Chia et al. 2018; Zhang et al. 2020; Valeta-Magara et al. 2019). In addition to migration, other CSC-derived factors profoundly influence the functional state of macrophages, by inducing their activation and polarization toward a pro-tumor phenotype. For instance, the cytokines IL-6 and IL-10 activate the transcription factor STAT3, which inhibits in macrophages the genes encoding for anti-tumor cytokines (Raghavan et al. 2019; Wyckoff et al. 2004; Wu et al. 2010; Kobatake et al. 2020; Gabrusiewicz et al. 2018). In glioblastoma, CSCs produce the immunosuppressive cytokine TGFβ which favors the functional polarization of pro-tumorigenic TAMs. Other immunosuppressive cytokines secreted by CSCs include IL-4 and IL-13, typically shifting to an M2-like phenotype (Chen et al. 2021; Clara et al. 2020; Taniguchi et al. 2020; Zhang et al. 2019b) (Fig. 1). Immune evasion is one of the major mechanisms by which CSCs can resist to the immune attack and survive. In general, CSCs express low levels of MHC molecules and of co-stimulatory receptors (e.g., CD80), which are necessary for optimal recognition by and triggering of immune cells. Instead, they express high levels of the checkpoint ligand PD-1L (Clara et al. 2020). In a recent study it has been reported that CSCs from squamous cell carcinoma upregulate the molecule B7-H3, another B7-family immune checkpoint also involved in T cell inhibition and evasion from immune surveillance (Wang et al. 2021). Another mechanism to hijack the immune control is to prevent phagocytosis via the inhibitory loop composed by the membrane molecule CD47 interacting with the protein Signal-regulatory protein alpha (SIRP1α) on phagocytic cells (Theocharides et al. 2012; Liu et al. 2017) (Fig. 2).

#### From the macrophage side

On the macrophage side, TAMs can support CSCs and their niche. Niche formation and maintenance is of paramount importance for CSC survival and renewal. TAM-derived factors implicated in biological processes such as Epithelial Mesenchymal Transition (EMT), maintenance of stemness features and more in general survival from stressful environment, include a number of cytokines, such as: IL-6, IL-1β, TNFα, TGFβ; chemokines: CCL2, CCL5, CCL8; matrix macromolecules and growth factors (Lu et al. 2014; Zhang et al. 2019b, 2020; Valeta-Magara et al. 2019; Guo et al. 2019; Huang et al. 2020; Wei et al. 2019a; Chen et al. 2019b) (Fig. 1). A recent paper demonstrated that the chemokine CCL2, produced by macrophages, support the expansion of CD44^+^ALDH1^+^ breast CSCs via activation of β-Catenin and increased expression of the transcription factors SOX2, OCT3/4 and NANOG (Zhang et al. 2021).

As mentioned above, TAMs are active producers of matrix-degrading enzymes and also of ECM macromolecules, thus contributing to the incessant remodeling of the tumor stroma (Liguori et al. 2011; Afik et al. 2016).

For instance, TAMs produce heparan sulfate proteoglycans and collagenous fibers, and may actually outnumber the fibroblasts, the canonical cells producing collagen types; furthermore, TAMs produce several matrix-related molecules, such as: fibronectin, the reactive truncated isoform of fibronectin called MSF (Migration Stimulation Factor), Osteopontin, and the matrix cross-linker enzyme F13a1 (coagulation factor XIII a1) (Liguori et al. 2011; Afik et al. 2016; Solinas et al. 2010).

Matrix components are crucial for preserving the niche architecture as well as for the communication between CSCs and the surrounding cells. In breast tumors, CSCs upregulate the enzyme hyaluronan synthase 2 (HAS2), which is important for the new synthesis of hyaluronic acid, a major polysaccharide component of the ECM. Okuda et al. demonstrated that HAS2^high^ tumor cells express a layer of pericellular hyaluronan that facilitates their attachment to TAMs, via the CD44 receptor. Through this interaction, CD44-expressing macrophages were stimulated to produce the growth factor PDGF-BB, which enhanced CSC self-renewal. This direct binding of hyaluronan-expressing CSCs to CD44 on TAMs provides an example of niche formation between the two cell types (Okuda et al. 2012; Kesh et al. 2020). Furthermore, using a mixed culture model of macrophages and CSCs, other studies demonstrated that macrophages actively stimulate HAS2 expression and hyaluronan production, thereby increasing CD44 engagement on tumor cells, and activate signaling pathways that are important for CSC maintenance (e.g. PI3K–4EBP1–SOX2). Thus, a reciprocal feed forward loop has been identified which includes the enzyme HAS2, the matrix component hyaluronan and signaling events from the CD44 receptor on CSCs and TAMs (Gomez et al. 2020; Skandalis et al. 2019) (Fig. 2).

Notably, the physical interaction between macrophages and CSCs appears crucial to support stemness features, as demonstrated in studies specifically addressing the importance of juxtacrine signaling mechanisms (Fig. 2). Cell–cell contact activates several pathways that are important for CSC, such as: SHH, NOTCH, STAT3 (Han et al. 2015; Hirata et al. 2014; Zhang et al. 2019c; Yang et al. 2013), PI3K/AKT, WNT/b-catenin, NANOG (Morgan et al. 2018; Wang et al. 2010; Wang et al. 2019; Wei et al. 2013; Zhang et al. 2019a) and NF-kB (Galoczova et al. 2018). The heterogeneity of tumor types underscores the diversity in crucial signaling pathways. For example, in the context of TAMs supporting stemness in colorectal cancer, the most prominent pathway is SHH (Jinushi et al. 2011), in pancreatic cancer is the TGFβ/ SMAD2/3/NANOG (Zhang et al. 2019b), and in hepatocarcinoma is the NOTCH pathway (Yang et al. 2013; Wang et al. 2016). A molecular mechanism of juxtacrine signalling between macrophages and stem cells has been elucidated in breast cancer. CSCs express the membrane GPI-anchored protein CD90 and the Ephrin type-A receptor 4 (EphA4); while CD90 functions as a bridge for adherence to the integrin CD11b on macrophages, the receptor EphA4, engaged by its ligand expressed by myeloid cells, activates the signaling pathways Src and NF-kB. Using CD90^high^ CSCs, the Authors reported that co-injection of tumor cells and macrophages into the mammary fat pad indeed promoted higher tumorigenicity in vivo and enhanced metastatic spreading (Lu et al. 2014).

Another transmembrane protein expressed by TAMs is the C-type lectin receptor CLEC4G, also named LSECtin. It has been reported that LSECtin interacts with CSCs via the Butyrophilin subfamily member A3 (BTN3A3) receptor, a member of the butyrophilin family. Their juxtacrine interaction is pivotal to drive tumor stemness (Liu et al. 2019). The relevance of macrophages within the CSC niche has been confirmed in macrophage-depletion experiments and by inhibiting their circulating precursors (monocytes). Antagonists of colony-stimulating factor 1 receptor (CSF1R) or of the chemokine receptor CCR2 substantially decreased the tumor-initiating properties of CSCs in pancreatic mouse tumor models (Mitchem et al. 2013). Also the inhibition of STAT3 and NF-kB in macrophages abolished the TAM-promoted stemness in several cancer types (Chen et al. 2021; Jinushi et al. 2011; Mitchem et al. 2013).

Soluble factors secreted by macrophages also play an important role in the support of CSC. Among the most relevant mediators are the growth factor MFG-E8 (milk-fat globule epidermal growth factor VIII), the immunomodulatory antimicrobial peptide hCAP-18/LL-37 and the Glycoprotein non-metastatic B (GPNMB) (Fig. 1). MFG–E8 is a secreted protein binding to phosphatidylserine and engaging the integrins αvβ3 and αvβ5. It regulates immune homeostasis through the phagocytosis of apoptotic cells, acting as a bridge molecule for the macrophages. MFG-E8-dependent recognition of apoptotic cells facilitates the tolerogenic activity of dendritic cells and induces the expansion of Foxp3 + T regulatory cells (Hanayama et al. 2004). Production of MFG-E8 by local macrophages is increased by GM-CSF secreted by mesenchymal cells. In the stem cell niche, MFG-E8 sustains CSC survival and functions to suppress host immune responses and to promote tumor angiogenesis (Jinushi et al. 2007, 2011; Keke et al. 2017). MFG-E8, in association with IL-6, induces STAT3 phosphorylation in CSCs and modulates the SHH pathway, critically impacting on the ability of stem cells to survive to chemotherapy drugs (Jinushi et al. 2011; Yang et al. 2013).

HCAP-18/LL-37 is an anti-microbial peptide secreted by phagocytes and epithelial cells with multiple functions. In addition to direct killing of pathogens, it regulates inflammatory responses and promotes wound healing by increasing the proliferation and migration of keratinocytes, as well as by stimulating neo-angiogenesis (Yang et al. 2020; Sabzevari et al. 2020). In pancreatic cancer, hCAP-18/LL-37 was strongly expressed by macrophages in response to tumor-derived Activin A, and increased CSC self-renewal, invasion, tumorigenicity, the expression of CD133 and of pluripotency-associated genes: KLF4, SOX2, OCT3/4 and NANOG. Mechanistically, hCAP-18/LL-37 was shown to bind and activate the formyl peptide receptor 2 (FPR2) and the P2X purinoceptor 7 receptor (P2X7R) (Sainz et al. 2015).

### Role of the protein GPNMB in the macrophages-CSC dialogue

Among the factors expressed by TAMs and inducing cancer cell stemness, the protein GPNMB occupies a special place. GPNMB is a highly glycosylated type I transmembrane protein that can be cleaved by proteases such as ADAM10. It is expressed in many cell types, such as osteoblasts, melanocytes, hepatocytes and leukocytes. This protein, also named Osteoactivin, has a vast array of biological activities, being involved in processes of cell adhesion and differentiation, tissue remodeling and repair after injury; some studies also reported that GPNMB limits inflammation and inhibits T cell-mediated immune responses (Abdelmagid et al. 2008; Safadi et al. 2001; Shikano et al. 2001; Haralanova-Ilieva et al. 2005; Ripoll et al. 2007; Kobayashi et al. 2019; Chung et al. 2020; Saade et al. 2021; Weterman et al. 1995; Singh et al. 2010).

GPNMB is also produced by several tumors. Substantial evidence indicates that GPNMB is implicated in disease progression in glioblastoma, melanoma and breast cancer (Kuan et al. 2006; Rose et al. 2007,2010). When expressed by tumor cells it is able to promote tumorigenesis, angiogenesis, cell invasion and metastasis (Zhou et al. 2015; Maric et al. 2013; Taya and Hammes 2018). Among immune cells producing GPNMB are macrophages and dendritic cells (Solinas et al. 2010; Chung et al. 2020; Yu et al. 2016). Our group reported that GPNMB is actively transcribed when macrophages are co-cultured with cancer cells (Solinas et al. 2010); furthermore, it is preferentially expressed by M2 macrophages (Liguori et al. 2021; Yu et al. 2016) and TAMs in mouse experimental tumors are positive for GPNMB (Liguori et al. 2021).

The first demonstration of a link between macrophage-derived GPNMB and stem cells was reported  in normal mesenchymal stem cells (MSCs) (Yu et al. 2016; Sondag et al. 2016). They demonstrated that GPNMB produced by macrophages stimulates the viability, proliferation and migration of MSCs. These effects were induced via engagement of the CD44 receptor and the activated ERK and AKT signaling pathways.

In mouse tumor models, we have recently reported that macrophage-derived soluble GPNMB triggers the expansion of cancer stem cells growing in vitro as self-renewing spheroids. These sphere-forming cells expressed markers of mesenchymal stem cells (e.g. CD199 and CD117 and Sca-1), as well as genes coding for stem cell transcription factors (e.g. *Nanog*, *Oct3/4*, *DNMT and Brachyury*) (Liguori et al. 2021). Similar results were demonstrated in GPNMB-transduced tumor cells that showed a remarkably high tumorigenicity and metastatic ability in vivo (Liguori et al. 2021). When endogenously produced by tumor cells from breast cancer and cholangiocarcinoma, GPNMB expression was associated with the ability to form spheroids in vitro containing elements with CSC properties (Raggi et al. 2017; Chen et al. 2018).

Maric et al. reported that the tumor-promoting effects of GPNMB were associated with elevated PI3K/AKT/mTOR signaling and increased β-catenin transcriptional activity (Maric et al. 2019). In our study we obtained evidence that the protein GPNMB also stimulates in cancer cells several other crucial pathways, such as MAPKs, AMPK and Src, in addition to STAT5 (Liguori et al. 2021). In primary methylcolantrene-induced fibrosarcoma, soluble GPNMB released by macrophages binds to the CD44 receptor on tumor cells and triggers the proliferation of CSCs. We further demonstrated that CD44 engagement by GPNMB activates in tumor cells the expression of several factors, including chemokines, e.g.: CXCL1, CXCL2, CCL2, CCL5, CCL7, cytokines: IL-6, IL-11, IL-33, and the IL-33 receptor: IL-1RL1, also named ST2 (Liguori et al. 2021).

The cytokine IL-33 is of particular interest in the context of cancer stemness, as a number of studies have been recently published. IL-33 is a relatively new member of the IL-1 cytokine family and it is expressed by epithelial cells, fibroblasts and immune cells (Dinarello 2005; Schmitz et al. 2005). IL-33 has many biological functions that are involved both in the regulation of adaptive immune responses (Schmitz et al. 2005; Schiering et al. 2014; Bonilla et al. 2012) and in tissue repair (Miller et al. 2008; Jones et al. 2010; Li et al. 2014). Its role in tumors is under debate; in fact, on the one hand IL-33 can promote T cell antitumor activity (Villarreal et al. 2014; Gao et al. 2015), on the other, its expression has been associated with metastasis in several cancer models (Jovanovic et al. 2014; Gillibert-Duplantier et al. 2012; Liu et al. 2014) and, accordingly, it is under study as a therapeutic target (Sun et al. 2021). Fang et al. observed that the overexpression or administration of IL-33 enhanced the growth of colon cancer cells, the formation of cell spheres, and the expression of stem cell genes (NANOG, NOTCH3 and OCT3/4) via phosphorylation of JNK (Fang et al. 2017). Furthermore, this cytokine is able to recruit macrophages at the tumor site and to trigger their production of PGE2. Activation of the JNK pathway was observed also in glioma, where IL-33 and its receptor IL-1RL1 were found overexpressed and were associated with increased cell migration, invasion, epithelial-mesenchymal transition, stemness features and poor patient survival (Lin et al. 2020).

Interestingly, two different polymorphisms in the IL-33 gene have been reported to increase the expression of IL-33 and to be associated with higher risk to develop hepatocellular carcinoma (Wei et al. 2019b; Pan et al. 2020). Like other factors involved in stemness, IL-33 is also capable of inducing resistance to chemotherapy. Lin et al. described that IL-33 prevents cancer cell death induced by temozolomide, a drug used in the clinic to treat glioma tumors (Lin et al. 2020). Furthermore, this cytokine is able to induce polyploidy; this event leads to the transformation of tumor cells into polyploid giant cells, which have an abnormal cell cycle, without cell division, accompanied by deregulation of SNAIL and inactivation of p53. This mechanism could be responsible for the failure of anticancer treatment (Kudo-Saito et al. 2020). Taniguchi et al. reported a mechanism linking IL-33 and cancer stemness that includes the activity of macrophages (Taniguchi et al. 2020). They found that IL-33 induces the accumulation and differentiation of macrophages expressing the IL-33 receptor IL-1RL1. Macrophages responding to IL-33 produce TGF-β that, in turn, promotes CSC invasion and drug-resistance (Taniguchi et al. 2020).

**Fig. 3:**
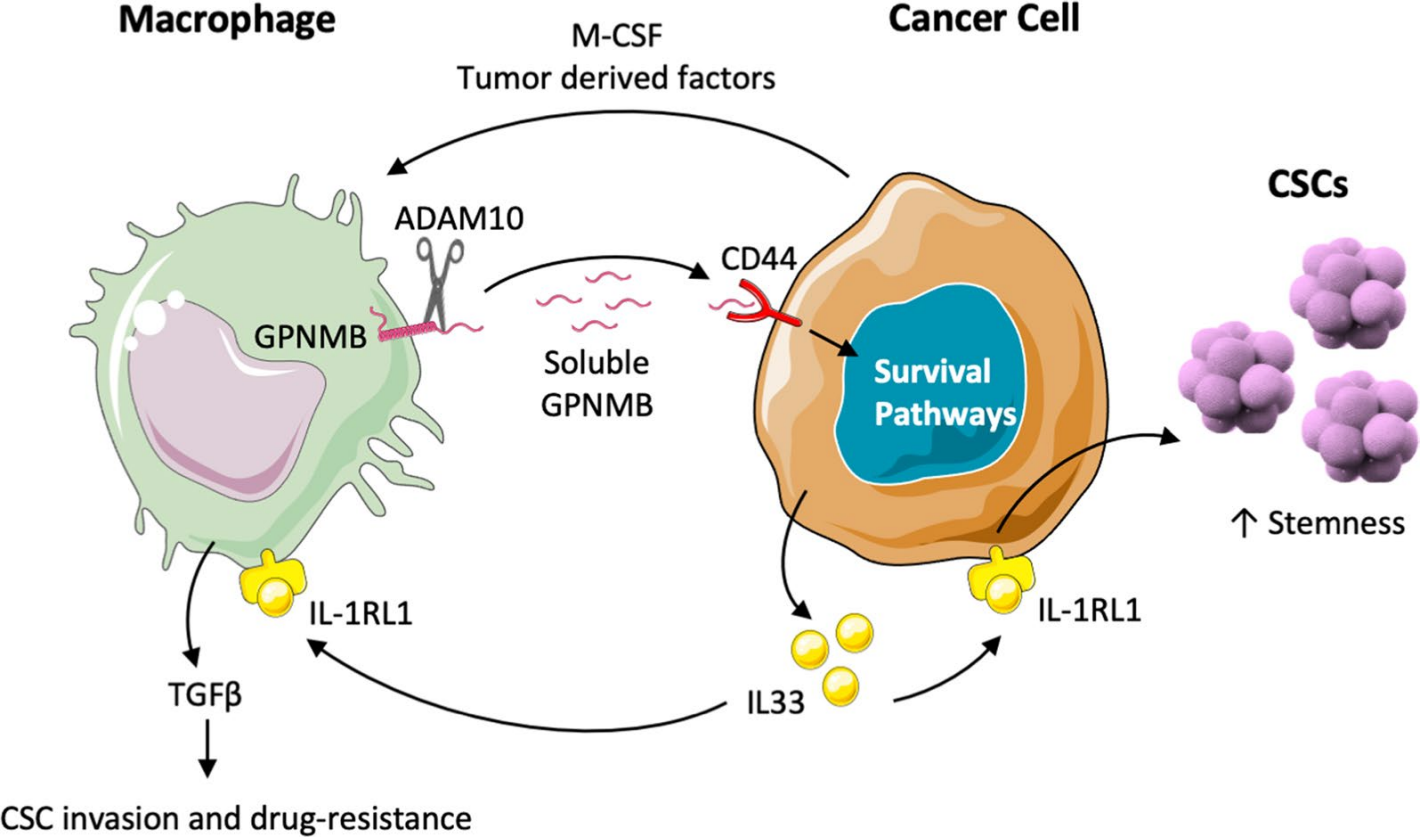
The soluble protein GPNMB produced by macrophages induces cancer stemness via CD44 and IL-33. Cancer cells stimulate in macrophages the production of GPNMB by M-CSF and tumor derived factors. GPNMB can be cleaved from the membrane by metalloproteinases, such as ADAM10. Soluble GPNMB binds to the CD44 receptor and triggers the release of IL-33. This cytokine, through the binding to its receptor IL-1RL1, is able to expand the population of CSCs, and to stimulate macrophages to produce TGFβ, promoting CSC invasion and resistance to drugs. This figure was made with Servier Medical Art templates, which are licensed under a Creative Commons Attribution 3.0. Unported License (https://smart.servier.com)

Therefore, we can envisage a feed-forward loop where cancer cells stimulate in macrophages the production of GPNMB that binding to the CD44 receptor on tumor cells triggers the release of IL-33. This cytokine is able to expand the population of CSCs, and also to stimulate macrophages to produce TGFβ, further reinforcing the number of CSCs and their stemness features (Fig. 3).

### Therapeutic strategies to target CSC and macrophages

CSCs are the ideal target of cancer therapy, because they are responsible for tumor initiation, distant spreading and disease recurrence. A major problem is that CSCs are inherently resistant to conventional therapies (chemotherapy and radiotherapy). Indeed, chemotherapy decreases tumor burden by killing the differentiated and proliferating cells, but often results in the enrichment of resistant CSCs in residual tumors. Among the pharmacological regimens to eliminate CSCs, combined treatment of chemotherapy, angiogenesis inhibitors, tyrosine kinase inhibitors and immunotherapy are currently being investigated (Chen et al. 2021; Clara et al. 2020; Corbet and Prieur 2020).

Approaches that target cancer cell metabolism are also studied. Metformin is an old drug widely used for type II diabetes; it is well-known that metformin has the capacity to remodulate metabolic pathways that are altered in cancer, and to inhibit specific chromatin-modifying metabolites. Research studies indicated an impact of metformin also on CSCs and suggested that epigenetic changes may increase the sensitivity of tumor cells to chemotherapy (Jones et al. 2021). In ovarian cancer patients, metformin inclusion in the treatment strategy significantly reduced the proportion of ALDH + CD133 + CSCs and improved the overall survival (Brown et al. 2020). Furthermore it has been indicated that metformin may also have effects on the immune populations of the tumor microenvironment, by reducing the density of macrophages and reprogramming their functional activities with increased phagocytosis (Wang et al. 2020).

A number of studies have tested inhibitors directed to specific oncogenic pathways that are associated with stemness and self-renewal, as mentioned above: Wnt/β-catenin, SHH, NOTCH and Hippo (Chen et al. 2021; Clara et al. 2020; Corbet and Prieur 2020). For instance, Wnt antagonists combined with paclitaxel effectively reduced the content of CSC and tumor growth in preclinical experiments with patient-derived xenografts (Fischer et al. 2017).

Glasdegib, an inhibitor of Hedgehog, has been approved by FDA and showed clinical activity in acute myeloid leukemia (Lainez-Gonzalez et al. 2021).

However, a note of caution must be highlighted: these developmental pathways and signaling circuits are also used by normal developing cells as well as immune cells, thus their targeting represents a challenge for their clinical use.

As anti-tumor immune responses have such an important impact on disease progression, some studies investigated the efficacy of strategies to boost the patient immune response using dendritic cell-based cancer vaccines loaded with peptides derived from CSC. However, the low expression of MHC I molecules renders CSC undetectable to T lymphocytes. To bypass the CSC intrinsic capability to evade the immune control, inhibitors to the histone deacetylase 6 have been employed to increase MHC molecules on CSC, in the attempt to maximize their recognition by T cells (Clara et al. 2020; Angelis et al. 2019).

Therapies directed to the immune microenvironment have gained momentum in the last decade and offer another possible option to target CSCs; due to the peculiar role that macrophages play in preserving the stem niche, these cells appear to be a preferential target. In the last two decades, several approaches to block or modulate the pro-tumor and immunosuppressive effects of TAMs have been tested in preclinical studies, and some of them are under clinical investigation (Belgiovine et al. 2020; Anfray et al. 2019; Xiang et al. 2021). Strategies aimed to stop the recruitment of circulating monocytes at the tumor or at the niche site, have employed inhibitors of chemokines and antagonists of the CSF1 receptor (Cassetta and Pollard 2018; Allavena et al. 2021). Although a significant decrease of macrophage influx in tumor tissues was observed, the anti-tumor efficacy was limited, probably because of the redundancy of the chemokine world (with many different ligands and shared receptors) and the continuous release of new myeloid progenitors from the bone marrow.

Cytokines, that are involved in the cross-talk between macrophages and CSC, and are of pivotal importance for the maintenance of their stemness status, can be pharmacologically targeted. For example, IL-6 signaling can be blocked by anti-IL-6 or IL-6R antibodies or by small-molecule inhibitors of the STAT3 pathway. Antagonists to the TGFβ pathway are also of considerable interest, in view of the pleiotropic effects of TGFβ on CSCs (Clara et al. 2020; Kim et al. 2021).

A different approach is to reprogram the functional activity of macrophages in the direction of anti-tumor effectors. It has long been known that when appropriately stimulated e.g.: with Toll-like receptor (TLR) ligands, immunostimulatory cytokines and agonist antibodies to activating receptors, macrophages can efficiently kill tumor cells (Mantovani et al. 2017; Allavena et al. 2021; Belgiovine et al. 2020; Fitzgerald and Kagan 2020; Ishikawa and Barber 2008; Maeda et al. 2019; Anfray et al. 2021;  Parker et al. 2004). Furthermore, their phagocytic activity of dead cancer cells is an important source of tumor antigens that can trigger T cell-mediated immune responses. Macrophage phagocytosis can be inhibited by the molecule CD47 expressed on cancer cell surface and binding to the protein SIRPα on TAMs. Anti-CD47 blocking antibodies can restore the activity of macrophages and proved to have significant efficacy in preclinical models (Theocharides et al. 2012; Liu et al. 2017; Weiskopf and Weissman 2015). Anti-CD47 antibodies are currently being studied in clinical trials in tumor patients with promising results, and it is conceivable they may enhance also the phagocytic killing of CSCs (Liu et al. 2017).

Macrophages express signaling receptors and molecular pathways that can activate the production of immunostimulatory cytokines and the direct killing of tumor cells. For example, activation of the CD40 receptor with agonist anti-CD40 mAbs, mimicking the natural ligand CD40L expressed by T cells, switches immunosuppressive TAMs into M1-like macrophages, re-establishing immune surveillance (Allavena et al. 2021; Huffman et al. 2020; Vonderheide 2020). Numerous clinical studies with anti-CD40 agonist mAbs are under way in patients with advanced tumors, in combination with chemotherapy or checkpoint immunotherapy.

Reprogramming of TAMs has been attempted also through the engagement of TLRs, for example TLR3, TLR7 and TLR8, specialized sensors of ectopic nucleic acids located in endosomal compartments. Engagement of TLRs by a number of available synthetic compounds triggers the transcription factor NF-kB and the production of several immunostimulatory cytokines, including type I IFN, the master regulator of anti-tumor and anti-viral immunity (Fitzgerald and Kagan 2020; McWhirter and Jefferies 2020). Another sensor of nucleic acids is STING (Stimulator of interferon genes), also leading to the production of IFNs (Ishikawa and Barber 2008). In several preclinical cancer models, stimulation of TLRs and of STING in immune cells successfully elicited antitumor immunity (Vanpouille-Box et al. 2019). On the basis of these results a number of TLR and STING agonists moved ahead for testing in cancer patients, most frequently in combination with other chemotherapeutic and immunotherapeutic regimens.

Although it is not clear whether reactivation of the cytotoxic and immunostimulatory properties of TAMs can be directed also against CSCs, it is reasonable to believe that acting on multiple fronts (i.e., hitting both the CSCs and the microenvironment) could be a winning solution. Furthermore, activation of type I IFN should increase the expression of MHC molecules, and therefore makes CSCs recognizable by the adaptive immunity. We can also envisage that, although CSCs are able to resist drug-mediated damage, pharmacological treatments could, in any case, have an impact by causing the upregulation of molecules belonging to a “stress signature”; these stress molecules may serve as activating ligands for cytotoxic immune cells, such as Natural Killers (Tallerico et al. 2016; Donini et al. 2021).

In conclusion, TAMs play an important structural and protective role in the niche where stem cells allocate; the potential of therapeutic strategies directed to TAMs is increasingly considered and a large amount of pre-clinical studies are now available. Combined approaches targeting both CSCs and the protective immune environment, macrophages in particular, are currently being investigated. The hope is that by removing the soil beneath their feet CSCs become more vulnerable and their complete eradication can be achieved.

## Data Availability

Not applicable.

## Abbreviations

ALDH1: Aldehyde dehydrogenase 1
ABCG2: ATP-binding cassette sub-family G member 2
BTN3A3: Butyrophilin subfamily member A3
CSCs: Cancer stem cells
CSF1R: Colony-stimulating factor 1 receptor
EphA4: Ephrin type-A receptor 4
GM-CSF: Granulocyte–macrophage growth factor
GPNMB: Glycoprotein non-metastatic B
HAS2: Hyaluronan synthase 2
M-CSF: Macrophage growth factor
MFG-E8: Milk-fat globule epidermal growth factor VIII
MSCs: Mesenchymal stem cells
OCT3/4: Octamer-binding transcription factor 3/4
SHH: Sonic Hedgehog
SIRP1α: Signal-regulatory protein alpha
SOX2: Sex determining region Y-box
TAMs: Tumor-associated macrophages
TGF β: Transforming growth factor β
